# Auricular Cartilage Regeneration with Adipose-Derived Stem Cells in Rabbits

**DOI:** 10.1155/2018/4267158

**Published:** 2018-03-18

**Authors:** Se-Joon Oh, Hee-Young Park, Kyung-Un Choi, Sung-Won Choi, Sung-Dong Kim, Soo-Keun Kong, Kyu-Sup Cho

**Affiliations:** ^1^Department of Otorhinolaryngology and Biomedical Research Institute, Pusan National University School of Medicine, Pusan National University Hospital, Busan, Republic of Korea; ^2^Department of Pathology, Pusan National University School of Medicine, Pusan National University Hospital, Busan, Republic of Korea

## Abstract

Tissue engineering cell-based therapy using induced pluripotent stem cells and adipose-derived stem cells (ASCs) may be promising tools for therapeutic applications in tissue engineering because of their abundance, relatively easy harvesting, and high proliferation potential. The purpose of this study was to investigate whether ASCs can promote the auricular cartilage regeneration in the rabbit. In order to assess their differentiation ability, ASCs were injected into the midportion of a surgically created auricular cartilage defect in the rabbit. Control group was injected with normal saline. After 1 month, the resected auricles were examined histopathologically and immunohistochemically. The expression of collagen type II and transforming growth factor-*β*1 (TGF-*β*1) were analyzed by quantitative polymerase chain reaction. Histopathology showed islands of new cartilage formation at the site of the surgically induced defect in the ASC group. Furthermore, Masson's trichrome staining and immunohistochemistry for S-100 showed numerous positive chondroblasts. The expression of collagen type II and TGF-*β*1 were significantly higher in the ASCs than in the control group. In conclusion, ASCs have regenerative effects on the auricular cartilage defect of the rabbit. These effects would be expected to contribute significantly to the regeneration of damaged cartilage tissue *in vivo*.

## 1. Introduction

Tissue engineering has been extensively used in the medical field, and recent studies are under way to regenerate cartilage in various disease conditions. Cartilage tissue engineering can offer a promising solution for restoring damaged cartilage and has the potential to overcome limitations of current treatments such as autologous cartilage grafting, reestablishing unique biological and functional properties of the tissue [1]. The identification of multipotential mesenchymal stem cells (MSCs) derived from adult tissues, including bone marrow stroma and a number of connective tissues, has provided exciting prospects for cell-based tissue engineering and regeneration [2]. Although bone marrow has been the main source for the isolation of multipotent MSCs, adipose tissue is another alternative source that can be easily obtained in larger quantities [3, 4].

MSCs have the ability to differentiate into chondrocytes in a nonvascularized area [5, 6]. Therefore, recent studies have focused on the differentiation of MSCs into chondrocytes for use in cartilage tissue engineering [7]. Among MSCs, adipose-derived stem cells (ASCs) have been recognized as an appropriate cell type with chondrogenic potential and high proliferative capacity [8–10]. Several studies showed that the chondrogenic process of ASCs could be successfully conducted using transforming growth factor- (TGF-) *β*1 and bone morphogenetic protein- (BMP-) 2 [11, 12]. Moreover, ASCs could be also easily harvested during a liposuction procedure with less discomfort and donor site morbidity [13]. However, little study has been performed for growth factors associated with auricular cartilage regeneration by ASCs.

The purpose of this study was to evaluate whether ASCs could differentiate into auricular cartilage and to know growth factors associated with regeneration of auricular cartilage defects by ASCs in rabbits.

## 2. Materials and Methods

### 2.1. Animals

Six-month-old female Dutch rabbits were purchased from Samtako Co. (Osan, Republic of Korea, http://www.samtako.co.kr) and bred in a specific pathogen-free animal facility. The animal study protocol was approved by the Institutional Animal Care and Use Committee of the Pusan National University School of Medicine.

### 2.2. Isolation and Culture of ASCs

Adipose tissue was obtained from the inguinal region of the rabbit. To isolate homologous ASCs, adipose tissue was washed extensively with equal volumes of phosphate-buffered saline (PBS) and digested with 0.075% collagenase type I (Sigma-Aldrich, St. Louis, MO) at 37°C for 30 min. Enzyme activity was neutralized with *α*-modified Eagle's medium (*α*-MEM) containing 10% fetal bovine serum (FBS), and the sample was centrifuged at 1200 ×g for 10 minutes to obtain a pellet. The pellet was filtered through a 100 *μ*m nylon mesh to remove cellular debris and then incubated overnight at 37°C with 5% CO_2_ in control medium (*α*-MEM, 10% FBS, 100 unit/mL penicillin, and 100 *μ*g/mL streptomycin). Following incubation, the plates were washed extensively with PBS to remove residual nonadherent red blood cells. The resulting cell population was maintained at 37°C with 5% CO_2_ in control medium. One week later, after the monolayer of adherent cells had reached confluence, cells were trypsinized (0.05% trypsin-EDTA; Sigma-Aldrich), resuspended in *α*-MEM containing 10% FBS, and subcultured at a concentration of 2000 cells/cm^3^. For the experiments, third- or fourth-passage ASCs were used.

Flow cytometric analysis was used to characterize the phenotypes of the ASCs. At least 50,000 cells (in 100 *μ*L PBS, 0.5% bovine serum albumin (BSA), and 2 mmol/L EDTA) were incubated with fluorescein isothiocyanate-labeled monoclonal antibodies against mouse stem cell antigen-1, CD44, CD90, CD45, CD117, and CD11b (Clontech, BD Biosciences, Palo Alto, CA) or with the respective isotype control. After washing, labeled cells were analyzed by flow cytometry using a FACSCalibur flow cytometer and CellQuest Pro software (BD Biosciences, San Diego, CA). The expression percentage of each marker of ASCs was determined by the percentage of positive events, as determined compared to the isotype-matched negative control.

ACSs were analyzed for their capacity to differentiate into adipogenic, osteogenic, and chondrogenic lineages, as described previously [14]. For adipogenic and osteogenic differentiation, cells were seeded in 6-well plates at a density of 20,000 cells/cm^2^ and treated for 3 weeks with adipogenic and osteogenic media. Adipogenic and osteogenic differentiation was assessed using Oil Red O staining, as an indicator of intracellular lipid accumulation, and Alizarin Red S staining, as an indicator of extracellular matrix calcification, respectively. Chondrogenic differentiation was induced using the micromass culture technique. Briefly, 10 mL of a concentrated ASC suspension (3 × 10^5^ cells/mL) was plated in the center of each well and treated for 3 weeks with chondrogenic medium. Chondrogenesis was confirmed by immunohistochemistry.

### 2.3. Subperichondrial Injection of ASCs

**Figure 1:**
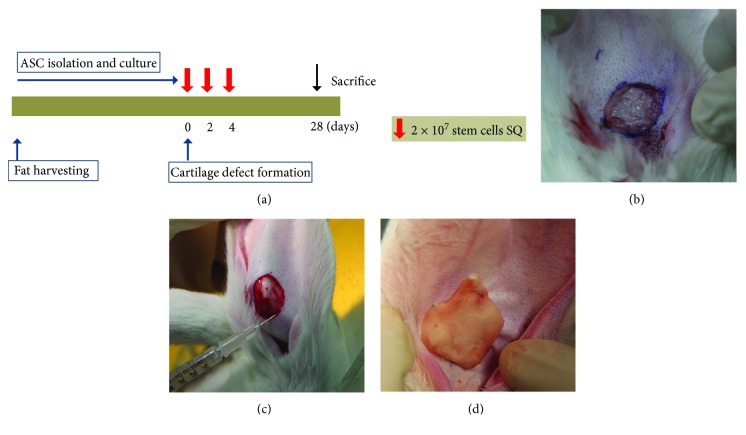
The experimental protocol and method. (a) Purified adipose-derived stem cells (ASCs; 2 × 10^7^ cells/mL) were injected at the site of the surgically created auricular cartilage defect subcutaneously (SQ) on days 0, 2, and 4. After auricular cartilage defect was created in the rabbit (b), ASCs were injected with a 26-gauze needle at the edge of the excision site (c) and the Spongostan was attached and sutured to the defect area (d).

The experimental protocol is summarized in Figure 1(a). Five Dutch rabbits were anesthetized with alfaxan (5 mg/kg) and 2% xylocaine (1 mg/kg). After shaving, we removed a 15 × 15 mm cartilage plate and skin in a circle from the midportion of each auricle with leaving the outer skin intact (Figure 1(b)). The right ear was treated with ASCs, and the left one was treated with PBS as the control ear.

ASCs were washed with PBS and suspended in PBS at a concentration of 2 × 10^7^ cells/mL. To evaluate the auricular cartilage regeneration effect of ASCs, 0.5 mL purified stem cells were injected with a 26-gauze needle at the edge of the excision site on postoperative days (POD) 0, 2, and 4 (Figure 1(b)). The Spongostan (Ferrosan, Copenhagen, Denmark) was attached and sutured to the defect area of both ears for preventing dryness, and the normal saline was frequently applied to maintain a constantly moist condition (Figure 1(c)).

### 2.4. Gross Evaluation of Cartilage Regeneration

Inspection for both ears of each animal was continued until complete closure of the auricular defect, or POD 28 in nonhealed cases. Photographs were taken with a digital camera (Nikon D40, Nikon, Japan) preoperatively, immediately postoperatively, and on POD 2, 4, and 6. After the seventh day, photographs were taken every other day up to POD 28 to determine the duration of auricular cartilage regeneration.

### 2.5. Histologic Analysis and Immunohistochemistry

Each animal was sacrificed at 4 weeks after ASC injection. After intraperitoneal injection of penobarbital (80 mg/kg), the auricles were resected and sent for histopathologic examination. The specimen was sectioned, and each section was stained with hematoxylin and eosin (H&E).

For Masson's trichrome staining, tissue sections were stained in Masson's composition solution for 5 min and differentiated in 5% phosphotungstic acid for 10 min. Tissue sections were then stained in aniline blue solution for 5 min, and excess stain was removed by washing with 0.2% acetic acid.

Immunohistochemistry for S-100 was performed as previously described [15]. For detection of S-100, sections were incubated with a polyclonal rabbit primary antibody against S-100 at a dilution of 1 : 1000 (Dako Z0311) at room temperature for 2 hours. Staining was revealed with Polymer-HRP (Dako EnVision®+ Dual Link System-HRP (DAB+) K4065) for 30 min. Incubation without the primary antibody was used as a negative control.

### 2.6. Quantitative Real-Time Reverse Transcription Polymerase Chain Reaction

For quantitative real-time polymerase chain reaction (qRT-PCR) analysis, total RNAs were extracted and reverse transcribed using random hexamers as previously described [16]. RT-PCR was performed using 10 ng of cDNA and SYBR Green mix (Bio-Rad Laboratories, Hercules, CA). The PCR primers used were as follows: collagen type II, forward 5′-CCCTGAGTGGAAGAGTGGAG-3′ and reverse 5′-GAGGCGTGAGGTCTTCTGTG-3′; TGF-*β*1, forward 5′-GGCAGTGGTTGAGCCGTGGA-3′ and reverse 5′-TGTTGGACAGCTGCTCCACCT-3′; insulin-like growth factor- (IGF-) 1, forward 5′-AGGAAGTACATTTGAAGAACGCAAGT-3′ and reverse 5′-CCTGCGGTGGCATGTCA-3′; and GAPDH, forward 5′-TCGACAGTCAGCCGCATCTTCTTT-3′ and reverse 5′-ACCAAATCCGTTGACTCCGACCTT-3′. The relative mRNA levels were evaluated using the 2^ΔΔC(t)^ method.

### 2.7. Statistical Analysis

All experiments were repeated at least in triplicate. Data are presented as mean ± SEM from all tissue specimens isolated. Statistical significance was assessed by Mann–Whitney *U* test using SPSS software package version 13.0 (SPSS Inc., Chicago, IL). A value of *p* < 0.05 was considered significant.

## 3. Results

### 3.1. MuItilineage Differentiation of ASCs

**Figure 2:**
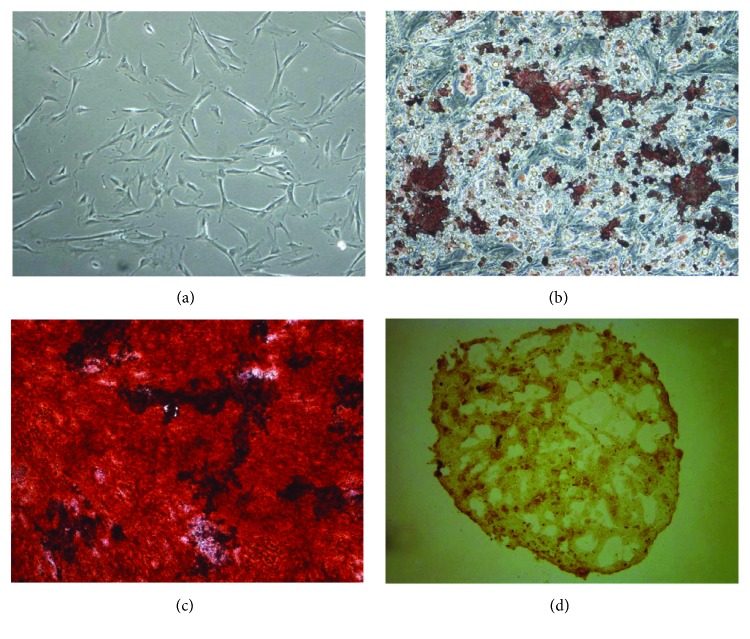
Characteristics of adipose-derived stem cells (ASCs). ASCs showed characteristics of mesenchymal stem cells in the fibroblast-like morphology appearance (a), adipogenesis (b), osteogenesis (c), and chondrogenesis (d) (magnification, 100x).

These putative ASCs had a spindle-shaped fibroblast-like appearance, similar to previous reported ASCs and bone marrow-derived MSCs (Figure 2(a)). Adipogenic differentiation was demonstrated by the accumulation of neutral lipid vacuoles by Oil Red O staining. A significant fraction of the cells contained multiple, intracellular lipid-filled droplets that stained with Oil Red O (Figure 2(b)). Osteogenic differentiation was confirmed by the deposition of Alizarin Red S-stained mineralized matrix. Calcification appeared as red regions within the cell monolayer (Figure 2(c)). Chondrogenic differentiation was confirmed by the formation of a sphere in micromass culture (Figure 2(d)).

### 3.2. Gross Findings

**Figure 3:**
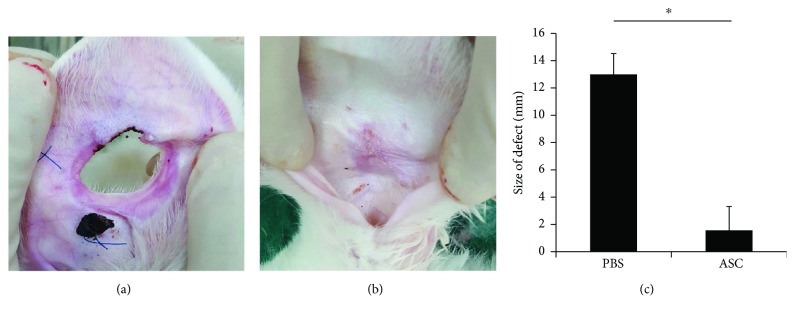
Gross findings of auricular cartilage defect. (a) The defect of control group remained thin around the perforation. (b) In the ASC-treated ears, the defects were completely repaired by chondrocytes with smooth surface and similar color with the surrounding tissue. (c) The defect size was significantly smaller in the ASC group than in the PBS group. Data are expressed as the mean ± SEM. ^∗^*p* = 0.008. ASCs, adipose-derived stem cells; PBS, phosphate-buffered saline.

There were no ears that showed signs of infection or graft rejection during the course of the study. The defect of the control group remained thin, presenting no chondrocyte proliferation around the perforation (Figure 3(a)). In the experimental group, after 4 weeks of ASC injection, the gross observation indicated that the cartilaginous defects were completely repaired by chondrocytes with smooth surface and similar color with the surrounding tissue (Figure 3(b)). Furthermore, we measured the lengths of the major and minor axes of the final defect size. The defect size was 13.0 ± 1.52 mm in the control group and 1.5 ± 1.78 mm in the experimental group, which showed statistical difference (*p* = 0.008) (Figure 3(c)).

### 3.3. Microscopic Findings

**Figure 4:**
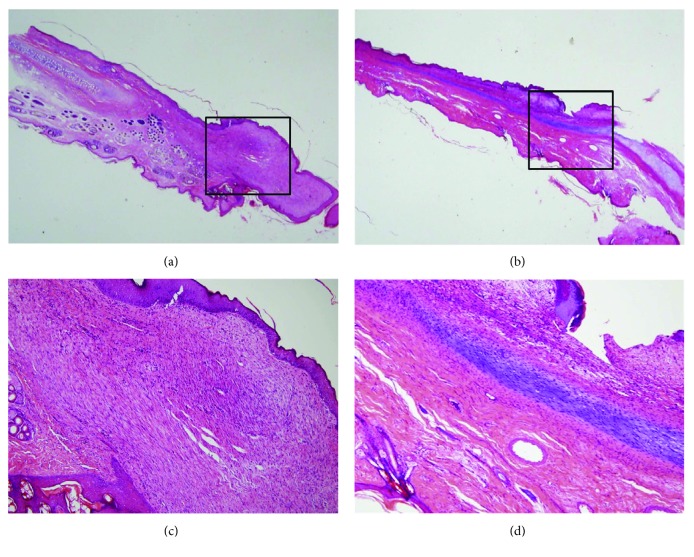
Histology of auricular cartilage defect. (a) Normal cartilaginous tissue was not visible in black square box in the control group. (b) New cartilage tissue was shown in black square box in the experimental group. (c) Fibrous tissue formation was observed instead of chondrocyte and extracellular matrix (ECM). (d) Typical cartilaginous features with chondrocytes, chondroblasts, and cartilage-specific ECM deposition were shown. Sections were stained with hematoxylin and eosin (magnification, a, b: 10x, c, d: 200x).

Although there was no new cartilage formation in the control group, fibrous tissue was observed on H&E stain (Figures 4(a) and 4(c)). In the experimental group, at 4 weeks after of ASC injection, typical cartilaginous features with chondrocytes, chondroblasts, and cartilage-specific extracellular matrix (ECM) deposition were shown. New cartilage was arranged in continuity with native cartilage remnant, filling the cartilage defect. Although mature normal cartilage shows obvious lacunae with dense chondrocytes and ECM, these samples showed a little lacuna with loosened structures (Figures 4(b) and 4(d)).

**Figure 5:**
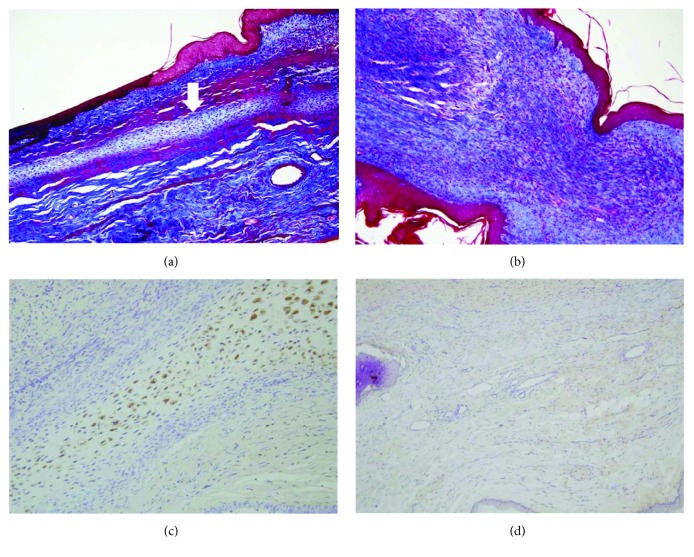
Masson's trichrome stain and immunohistochemistry. Masson's trichrome staining showed pale blue-colored cartilage formation (arrow) in the experimental group (a) and only collagenous tissue in subepithelial region of the control group (b). Imunohistochemistry for S-100 showed strongly stained chondrocytes and chondroblasts in the experimental group (c), but not in the control group (d) (magnification, 200x).

In the Masson's trichrome stain for collagen fibers, the chondroid matrix and cellular components were detected in the ASC group, although fibrosis and scar formation were shown in the control group (Figures 5(a) and 5(b)). Imunohistochemistry for S-100 showed strongly stained chondrocytes and chondroblasts in the ASC group, but not in the control group (Figures 5(c) and 5(d)).

### 3.4. Gene Expression of Collagen Type II and Growth Factors

**Figure 6:**
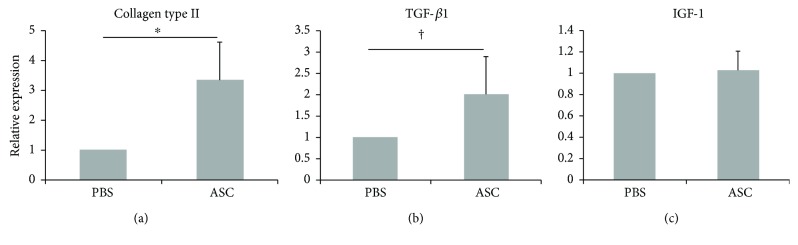
Expression of collagen type II, TGF-*β*1, and IGF-1. The relative expression of collagen type II (a) and TGF-*β*1 (b) were significantly higher in the ASC group than in the PBS group. However, there was no significant difference in the expression of IGF-1 between the ASC and PBS groups (c). Data are expressed as the mean ± SEM. ^∗^*p* = 0.008 and ^†^*p* = 0.016. ASCs, adipose-derived stem cells; PBS, phosphate-buffered saline.

The relative expression of collagen type II, TGF-*β*1, and IGF-I were 3.33 ± 1.28, 2.01 ± 0.51, 1.03 ± 0.18, respectively. The expression of collagen type II and TGF were significantly higher in the ASC group than in the PBS group (*p* = 0.008 and *p* = 0.016, resp.). However, there was no significant difference in the expression of IGF-1 between the ASC and PBS groups (*p* = 0.690) (Figure 6).

## 4. Discussion

Stem cell-based therapies for the repair and regeneration of various tissues and organs offer a paradigm shift that may provide alternative therapeutic solutions for a number of diseases. Much research has focused on MSCs which have shown to possess adipogenic, osteogenic, chondrogenic, myogenic, and neurogenic potential *in vitro* [13]. Therefore, MSCs represent an exciting progenitor cell source for application of cartilage tissue engineering and regenerative medicine. Although bone marrow has been the representative cell source, adipose tissue is ubiquitous and easily obtainable in larger quantities with little discomfort, implying that it may be an alternative source of MSCs for mesenchymal tissue regeneration and engineering [2, 13].

Auricular cartilage defect shows a poor capacity for self-repair because of a lack of dominating blood supply. Nevertheless, cartilage has become an important target for tissue engineering, not only because of the low degree of vascularization but also because of immune privilege and a lymphatic structure surrounded by dense ECM that is impervious to leukocytes [17]. Previous study showed successful repair of ear cartilage defect with chondrocytes induced from allogeneic bone marrow-derived MSCs in rabbits [18]. A human-ear-shaped cartilage was successfully constructed by cotransplantation of autologous microtia chondrocytes and bone marrow-derived MSCs into the preshaped biodegradable ear scaffold [19]. Although ASCs can proliferate and differentiate into chondrocytes, enabling chondrogenesis and cartilage defect repair [20], it remains unclear which of growth factors is associated with auricular cartilage regeneration by ASCs. Therefore, this study was performed to clarify the regenerative effects of ASCs on auricular cartilage defect by gross observation, histologic analysis, immunohistochemistry, and qRT-PCR for growth factors.

The present study presented the accelerated repair of auricular cartilage defect in ASC-treated rabbits. The regeneration of the auricular cartilage in these rabbits was markedly improved compared with that of normal controls, and the healing improvement was histopathologically characterized by new cartilage formation composed of chondrocytes and cartilage-specific ECM at the site of the surgically created defect. These findings were confirmed by Masson's trichrome stain that demonstrated pink nuclei subjacent to the pale blue staining chondroid matrix and fine fibrous tissue network showing intense metachromasia that divided the chondrocytes into clusters. Furthermore, ASCs differentiated to a chondroblastic phenotype with stronger expression of S-100 protein in the immunohistochemical staining and higher expression of type II collagen in qRT-PCR.

The TGF-*β* superfamily of proteins and their members such as BMP, fibroblast growth factors, and IGF are well-established regulatory factors in chondrogenesis [2]. As the cartilage repair process progresses during cell therapy with MSCs, the influence of TGF-*β* superfamily created by the host tissue on the transplanted MSC should be considered [21]. The TGF-*β* superfamily is one of the most investigated and biologically active substances within the field of cartilage tissue engineering [22]. TGF-*β*1 was initially used for *in vitro* culture and can stimulate the synthetic activity of chondrocytes [23], although TGF-*β*3 has recently been shown to have the highest chondrogenic potential of all isoforms, and their action results in rapid cell differentiation [24, 25]. Furthermore, treatment of MSCs with TGF-*β*1 enhanced the synthesis of sulfated GSG and induced production of cartilaginous ECM [26]. In this study, the expression of TGF-*β*1 was significantly increased on the transplanted ASCs. Therefore, it could be concluded that the stem cell itself also promoted TGF-*β*1 expression, which was helpful for differentiation of chondrocytes.

IGFs play an important role in skeletal development. They stimulate cell proliferation and regulate apoptosis and expression of chondrogenic markers. Although the previous study reported that the expression of IGF-1 and TGF-*β*1 increased in the initial phase following acute cartilage wounding [27], the expression of IGF-1 was not increased in this study. These contradictory results may be due to the difference in the cartilage maturation. Because the role of IGF-1 in cartilage regeneration is mainly required to maintain cartilage integrity [28], cartilage maturation by ASCs in the present study might not be as enough as that of mature normal cartilage due to short study period.

It is unclear whether ASCs injected into a surgically created auricular cartilage defect divide, differentiate, and give rise to new ASC-derived chondrocytes, or whether they just orchestrate regeneration by secreting bioactive paracrine factors stimulating host cells to promote regeneration. Recent study shows that intra-articularly injected MSCs contribute to regeneration of articular cartilage in full-thickness cartilage defects mainly via a nonprogenitor-mediated mechanism using *in vivo* cell tracking model [29]. However, further studies are required to fully characterize the mechanism of ASCs in auricular cartilage regeneration, whether by direct differentiation of ASCs or by secretion of crucial mediators of ASCs.

The limitation of our study was relatively the short study period of 4 weeks. Although the four-week period was sufficient to confirm regeneration and wound healing of auricular cartilage defect, it was insufficient to observe the maturity of regenerated cartilage. Further long-term studies are required to clarify the regenerative effect of ASCs on auricular cartilage defect and the maturity of differentiated cartilage.

The present study showed that ASC treatment has a regenerative effect on auricular cartilage defect in rabbits, which is characterized by new cartilage formation composed of chondrocytes and cartilage-specific ECM at the site of the surgically created defect with stronger expression of S-100 protein and higher expression of type II collagen and TGF-*β*1.
